# Quantum Chemical Studies on the Prototropic and Acid/Base Equilibria for 2-Aminopyrrole in Vacuo—Role of CH Tautomers in the Design of Strong Brønsted Imino N-Bases

**DOI:** 10.3390/molecules30102112

**Published:** 2025-05-09

**Authors:** Ewa Daniela Raczyńska, Pierre-Charles Maria, Jean-François Gal

**Affiliations:** 1 Department of Chemistry, Warsaw University of Life Sciences (SGGW), ul. Nowoursynowska 159c, 02-776 Warsaw, Poland; 2 Institut de Chimie de Nice, University Côte d’Azur, UMR 7272 CNRS, Parc Valrose, 06108 Nice, France; pierre-charles.maria@univ-cotedazur.fr (P.-C.M.); jean-francois.gal@univ-cotedazur.fr (J.-F.G.)

**Keywords:** 2-aminopyrrole, tautomers, proton-transfers, gas-phase basicity/acidity, superbase-design origin, DFT studies

## Abstract

In the quest of the pivotal origin of the very strong gas-phase proton basicity for some iminopyrrole derivatives, proposed in the literature on the basis of quantum chemical calculations, the full tautomeric and acid/base equilibria were investigated in vacuo for 2-aminopyrrole exhibiting enamino–imino tautomerism. Thermochemistry of these processes investigated at the Density Functional Theory (DFT) level indicates a lower stability for the imino than for the enamino tautomers. However, the imino N atom in the imino forms displays an exceptionally high basicity, particularly in the minor and rare tautomers containing at least one tautomeric proton at the pyrrole C atom. This explains why derivatives of CH tautomers (being free of prototropy) display exceptionally high gas-phase proton basicity. As predicted by the Maksić group using quantum chemical methods, these derivatives can be considered as good organic imino N-superbase candidates. Unfortunately, some other structures of iminopyrrole derivatives (proposed by the same group) possess labile protons, and, thus, exhibit prototropy, resulting in the transformation into the more stable but less basic aminopyrrole derivatives under synthesis conditions or acid/base equilibria measurements.

## 1. Introduction

Organic compounds containing the five-membered ring made up of four C-sp^2^ and one N atom, called the pyrrole system, have drawn particular attention of chemists, biochemists, microbiologists, and pharmacologists because of their presence in many biologically active structures (see for example Refs. [1,2,3,4,5,6,7,8]). Pyrrole itself is not found in living organisms, but its cyclic subunit can be found in the skeleton of numerous molecules found in living organisms. For example, it is present in bicyclic 1*H*-indole (2,3-benzopyrrole) and its derivatives such as the amino acid L-tryptophan, the biogenic amine tryptamine, the neurotransmitter serotonin, and the hormone melatonin [9,10,11,12]. The pyrrole scaffold appears also in the structures of porphyrins of heme, chlorophylls, chlorins, porphyrinogens, vitamin B12, bile pigments (bilirubin and biliverdin), and also alkaloids isolated from various marine organisms, insects, fungi, or bacteria [3,13,14]. The pyrrole ring is a building part of numerous natural and synthetic products that has found diverse pharmacological applications [1,2,3,4,5,6,7,8]. Natural and synthetic pyrrole systems display anti-bacterial, anti-fungal, anti-protozoal, insecticidal, anti-viral, anti-malarial, anti-tubercular, anti-inflammatory, anti-depressant, anti-convulsant, anti-ulcer, anti-diabetic, anti-hypertensive, anti-psychotic, anti-cancer, anti-HIV activity, etc. [1,2,3,4,5,6,7,8]. Some of them have been applied as lipid-lowering, cytotoxic, anti-Parkinson, and anti-Alzheimer agents, enzyme inhibitors (e.g., HMG-CoA reductase, histone deacetylase, DPP IV, and GSNOR), and receptor antagonists (e.g., mGluR1, GnRH, dopaminergic, progesterone, and androgen) [1,4].

Because of these biological activities and pharmacological applications of the systems containing the pyrrole ring, detailed examinations of intra- and intermolecular proton-transfer processes are required to well describe structure–activity relationships. For example, the acid/base properties of the parent compound have already been investigated by various experimental techniques in different environments. Unsubstituted pyrrole exhibits exceptionally weak Brønsted acidity and basicity in solution [9,15,16,17,18], as well as in the gas phase [19,20,21,22]. However, adding various “pushing” (electron-donating) and “pulling” (electron-accepting) substituent groups on the pyrrole ring has led to some interesting derivatives, which can be classified as strong organic N-bases in the gas phase [23,24]. 

**Figure 1:**
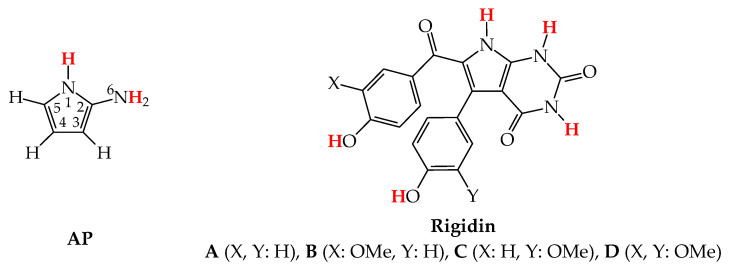
Structures of 2-aminopyrrole (**AP**, investigated in this work) and marine alkaloids (**rigidin A**, **B**, **C**, and **D**, containing **AP** part). Labile (tautomeric) protons are indicated in bold red. The numbering of heavy atoms is noted in the **AP** formula.

With the aim to unravel the origins of very strong gas-phase proton basicity of the push–pull pyrrole derivatives, we chose here the simplest molecule, containing one pushing amino group at the 2-position, 2-aminopyrrole (**AP** in Figure 1 and hereafter). This compound and some of its derivatives (substituted at N1, C3, C4, and/or C5) belong to the family of tautomeric heterocycles, like other azoles [25,26,27,28,29]. Some tautomers of neutral and protonated **AP** have already been investigated [26,27,28,29], but there is no complete examination on the thermochemistry of all possible intra- and intermolecular proton-transfers in **AP**, nor on its acidity/basicity parameters. Nevertheless, we can find numerous articles in the literature on the synthesis and experimental structure-confirmations of **AP** and its derivatives published in the last thirty years (see for example Refs. [27,28,29,30,31,32,33,34,35]). Although 2-aminopyrrole itself is not sufficiently stable, it can be preserved for a long time in the form of its protonated salts, e.g., trifluoroacetate, perchlorate, or tetraphenylborate, and can be easily isolated just before an experimental investigation from one of its salts by adding one or more equivalents of an organic base, e.g., triethylamine [31]. For example, substituted 2-aminopyrroles have been successfully used for the syntheses of the marine alkaloid rigidins A, B, C, and D [36,37]. Their general formulae are shown in Figure 1. Since they may display prototropy [25,38,39], all labile (tautomeric) protons are indicated in this figure.

Considering the scarcity of the literature data on the proton-transfer processes for 2-aminopyrrole, the complete tautomeric and acid/base equilibria in vacuo have been studied during this work, employing quantum chemical calculations. Contrary to the experimental techniques, which detect mainly major and some minor isomers (with percentage contents >1%), only theoretical methods give the opportunity to study all possible isomeric forms and all possible proton-transfer equilibria. In this way, thermochemistry of the complete proton-transfer processes could be examined for 2-aminopyrrole. Additionally, the detailed analysis of proton transfers for **AP** could shed some light on the origins of superbasicity in iminopyrrole derivatives, reported by the Maksić group [23,24], and signaled by us in Ref. [40]. 

## 2. Results and Discussion

### 2.1. Possible Prototropic Tautomers for Neutral, Deprotonated, and Protonated **AP**

**Figure 2:**
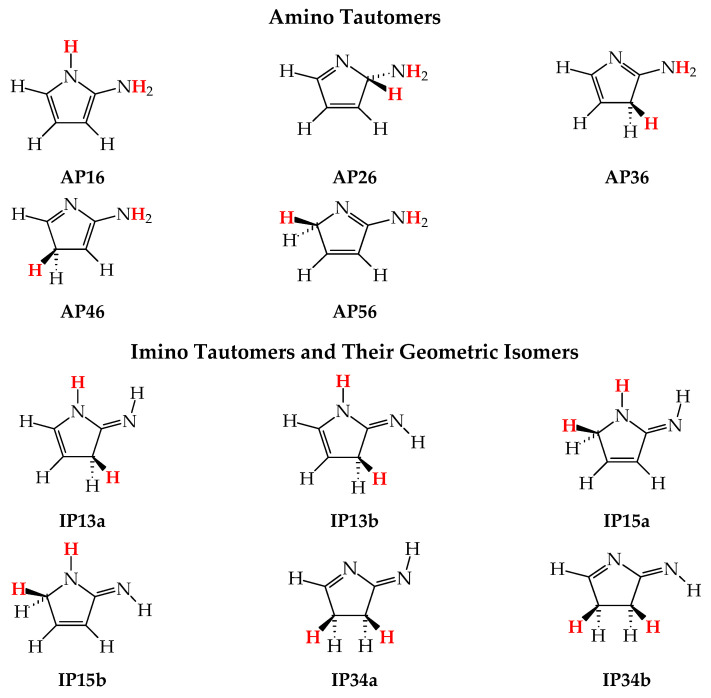
The eleven possible prototropic isomers for neutral 2-aminopyrrole. Labile (tautomeric) protons are indicated in bold red.

Neutral 2-aminopyrrole (**AP**) contains two labile (tautomeric) protons, one proton attached to the endo N1 atom, and the other proton linked to the exo N6 atom (Figure 1). Each of them can move between the n-π conjugated sites (N1, C2, C3, C4, C5, and/or N6; see Scheme S1 in Supplementary Materials). Hence, we can distinguish five amino tautomers (**AP16**, **AP26**, **AP36**, **AP46**, and **AP56**), analogous to those for tautomeric unsubstituted pyrrole, and additionally three imino tautomers (**IP13**, **IP15**, and **IP34**), in which the exo amino group is engaged in the tautomeric conversions (Figure 2). Note that the figures in the bold labels of isomers correspond to the positions of the tautomeric protons. Two extreme isomers for each imino form, one containing a synperiplanar (**a**) and the other one having an antiperiplanar imino H atom (**b**) vis-à-vis N1, arise from the configurational (geometric) isomerism about the C2=N6 double bond (see Scheme S2 in Supplementary Materials). Consequently, eleven isomers can be written for neutral 2-aminopyrrole. The geometric isomers (**a** and **b**) can be called “rotamers” of the exo C2=N6H group.

In all tautomeric rearrangements, π-electrons move in concert with the proton transfers. According to the Pauling definition of prototropy [38], all prototropic tautomers of 2-aminopyrrole are written without separation of the positive and negative charges, as displayed by the **AP** resonance structures (see Scheme S1 in Supplementary Materials). In the amino tautomer **AP16**, the labile protons are at the N1 and N6 atoms, and we call this the NHNH form. The amino tautomers **AP26**, **AP36**, **AP46**, and **AP56**, and also the imino isomers **IP13a**, **IP13b**, **IP15a**, and **IP15b**, possess the labile protons at the N (N6 or N1) and C atoms (C2, C3, C4, or C5). They can be named CHNH forms. In the last imino isomers **IP34a** and **IP34b**, the labile protons are at the C3 and C4 atoms, and we call them the CHCH forms. Note that our analysis for the first time refers to the complete tautomeric mixture of eleven prototropic isomers of 2-aminopyrrole. Dewar, De Rosa, and their co-workers [26,27] analyzed only one amino form (**AP16**) and two imino tautomers (**IP13** and **IP15**) in their studies on tautomerism for neutral 2-aminopyrrole. Other possible tautomers were ignored. It is not clear why only three tautomers were studied, and we complete here the previously reported results.

**Figure 3:**
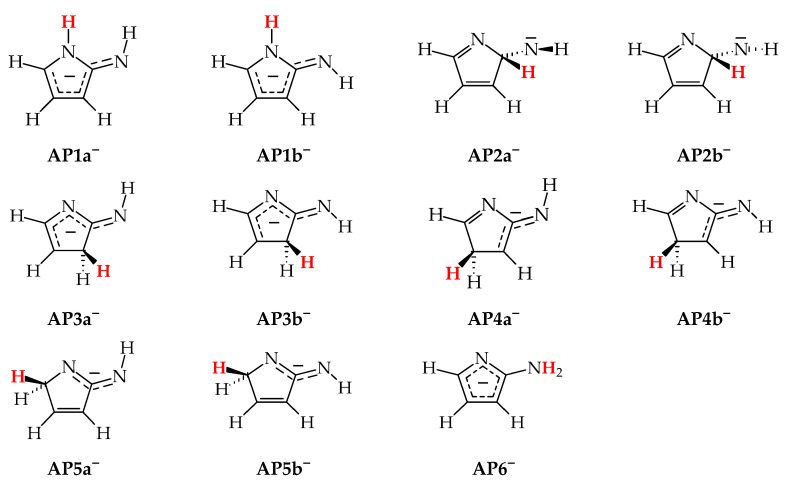
The eleven prototropic isomers possible for mono-deprotonated 2-aminopyrrole. The labile (tautomeric) proton is indicated in bold red.

The mono-deprotonated **AP** isomers are formed by loss of one labile proton in the amino and imino isomers of the tautomeric mixture of the neutral derivative. This process leads to the tautomeric mixture **AP^−^** containing the mono-anionic isomers, which now contains only one tautomeric proton. Mono-deprotonation of the amino tautomers **AP16**, **AP26**, **AP36**, **AP46**, and **AP56** at the N1, C2, C3, C4, and C5 atoms, respectively, gives one common mono-anionic isomer **AP6^−^** possessing the labile proton at N6 (Figure 3). In this reaction, the **AP** amino isomers can alternatively lose one labile proton at the amino N6 atom {in syn- (**a**) or anti-periplanar (**b**) position vis-à-vis N1}, leading to the corresponding isomers of the mono-deprotonated form: **AP1a^−^**, **AP1b^−^**, **AP2a^−^**, **AP2b^−^**, **AP3a^−^**, **AP3b^−^**, **AP4a^−^**, **AP4b^−^**, **AP5a^−^**, and **AP5b^−^**. The isomers **AP1a^−^** and **AP1b^−^** can also be formed after mono-deprotonation of the imino isomers **IP13a**, **IP13b**, **IP15a**, and **IP15b** at the C3 and C5 atom, respectively. In turn, mono-deprotonation of **IP13a**, **IP13b**, **IP15a**, and **IP15b** at N1 gives **AP3a^−^**, **AP3b^−^**, **AP5a^−^**, and **AP5b^−^**, respectively, while for **IP34a** and **IP34b** at C3 it leads to **AP4a^−^** and **AP4b^−^**, and for **IP34a** and **IP34b** at C4 it leads to **AP3a^−^** and **AP3b^−^**. To our knowledge, the eleven isomers of mono-anionic 2-aminopyrrole (**AP^−^**) have not yet been reported in the literature, and this is the first complete analysis of its tautomeric mixture.

**Figure 4:**
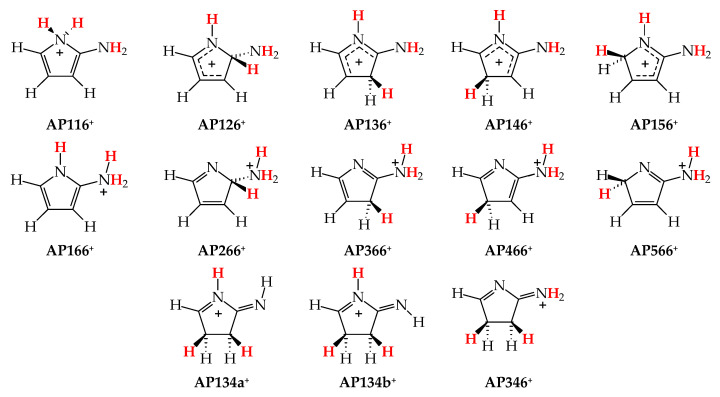
The thirteen prototropic isomers considered in this work for mono-protonated 2-aminopyrrole. Labile (tautomeric) protons are indicated in bold red.

When the tautomeric mixture of neutral 2-aminopyrrole (**AP**) gains one proton in the mono-protonation reaction, the mono-cationic tautomeric mixture is formed **(AP^+^**). The six mono-cationic isomers **AP116^+^**, **AP126^+^**, **AP136^+^**, **AP146^+^**, **AP156^+^**, and **AP166^+^** can be derived by mono-protonation of the neutral amino tautomer **AP16** at N1, C2, C3, C4, C5, and N6, respectively (Figure 4). Alternatively, the four tautomers **AP126^+^**, **AP136^+^**, **AP146^+^**, and **AP156^+^** can be formed when one proton is gained by N1 in the tautomers **AP26**, **AP36**, **AP46**, and **AP56**. The stability of **AP266^+^**, **AP366^+^**, **AP466^+^**, and **AP566^+^**, mono-protonated at N6, can also be verified. The mono-protonation of the two isomers **a** and **b** of the imino tautomers **IP13**, **IP15**, and **IP34** at N6 leads to the common mono-cations **AP136^+^**, **AP156^+^**, and **AP346^+^**, respectively. The two isomers **a** and **b** of the imino tautomer **IP34** can be also mono-protonated at N1, leading to the isomeric pair **AP134a^+^** and **AP134b^+^**. Consequently, each of the thirteen possible isomers of the mono-cationic form **AP^+^** contains three labile (tautomeric) protons and displays prototropy. In the literature, we found two theoretical reports on prototropic tautomerism in mono-protonated 2-aminopyrrole. De Rosa and co-workers [27,28] discussed only three mono-cationic tautomers, **AP136^+^**, **AP156^+^**, and **AP166^+^**, referring to the mono-protonation of the amino isomer **AP16** at C3, C5, or N6, respectively. Regarding this tautomerism in vacuo, the authors applied quantum chemical calculations at diverse levels of theory {HF, MP2, MP4SDQ, QCISD, and QCISD(T)} using various basis sets, but their analyses gave an incomplete picture of the tautomeric mixture of **AP^+^**.

### 2.2. Thermochemistry of Prototropic Conversions for **AP**, **AP^−^**, and **AP^+^**

Quantum chemical calculations were carried out for possible tautomers/rotamers of neutral and ionic forms of 2-aminopyrrole using the DFT (Density Functional Theory) method [41] with the B3LYP (Becke three-parameter hybrid exchange functional and non-local correlation functional of Lee, Yang, and Par) functional [42,43] and the 6-311+G(d,p) basis set with polarization and diffuse functions [44]. This level of theory has been proposed by Koppel, Leito, Maksić and their co-workers as adequate for investigating the thermochemistry of proton-transfer processes in the gas phase, as well as for estimations of the gas-phase proton acidity/basicity parameters [24,45,46]. All isomers given in Figure 2, Figure 3 and Figure 4 were optimized without symmetry constraint and their energy minima (with positive frequencies) calculated at the DFT(B3LYP)/6-311+G(d,p) level. Their atom coordinates, electronic energies, and thermochemical data are included in Tables S1 and S2, respectively (Supplementary Materials). The mole-fractions of all isomers (*x*_i_) in the tautomeric mixtures of neutral and ionic 2-aminopyrrole were estimated using the relative Gibbs energies (Δ*G*s) calculated at 298.15 K as the difference between a given isomer and the most stable one. For calculations, Equation (1) was employed.

*x*_i_ = exp{−Δ*G*(**AP**_i_, **AP**_i_^−^, or **AP**_i_^+^)/*RT*}/{Σ_1_^n^exp[−Δ*G*(**AP**_i_, **AP**_i_^−^, or **AP**_i_^+^)/*RT*]} (1)

As was expected, the amino tautomer **AP16** (Figure 2) is the most stable form {displays the lowest Gibbs energy (*G*)} for neutral 2-aminopyrrole. The other amino and imino tautomers possess higher *G* values. Their relative Gibbs energies (Δ*G*) are between 20 and 80 kJ mol^−1^ (Table 1). The participation of some of them in the tautomeric mixture of **AP** can be significant, and thus they can be considered in the acid/base equilibria. The Mezey et al. rule [47,48], used in quantum chemical stability-estimations, helps us to select important isomers. This rule was derived about forty-five years ago for prototropic tautomers of the nucleobases guanine and adenine, and for their protonated forms. Authors used the methodology and ab initio levels of quantum chemical calculations suitable at that time. Although quantum chemical methods and computers have progressed since that time, we can still use this rule for tautomeric systems, because tautomeric equilibria are very sensitive to environment, and tautomeric preferences can change, for example, when going from the gas phase through various solutions to the solid state, as well as from neutral to ionized forms [25,39,49]. According to the Mezey rule, the lowest limit of significant relative Gibbs energies between the favored and other isomers in the isomeric mixture cannot be higher than 40 kJ mol^−1^. Employing this rule, we can neglect only three isomers in the isomeric mixture: two amino forms (**AP46** and **AP26**) and one imino isomer (**IP34b**). On the basis of our DFT-calculated Δ*G* values (<40 kJ mol^−1^), the stability order of the remaining eight isomers is as follows: **AP16** > **AP56** > **AP36** > **IP13b** > **IP34a** > **IP15b** > **IP13a** > **IP15a**. 

Note that some imino isomers (**IP13b**, **IP34a**, and **IP15b**) exhibit Δ*G*s close to those of the amino ones (**AP56** and **AP36**). This means that various types of prototropic conversions between the conjugated atoms of the pyrrole ring, as well as between the conjugated exo N6 atom and endo C atoms, could be detected in the tautomeric mixture of neutral **AP**. Moreover, regarding the Δ*G* values of the configurational isomers **a** and **b** for the imino tautomers **IP13**, **IP34**, and **IP15**, we can draw an additional observation. The favorable interactions between the functional groups, i.e., between N1H and the n-electron pair of N6 in **IP13b** and **IP15b**, and between N6H and the n-electron pair of N1 in **IP34a**, decrease the Δ*G* values by 4.4, 3.2, and 14.1 kJ mol^−1^, respectively, in comparison to those of their rotamers with the unfavorable interactions, i.e., repulsion between N1H and N6H in **IP13a** and **IP15a**, and repulsion between the n-electrons pairs of N1 and N6 in **IP34b**. This observation indicates without any doubt that the effects of geometric isomerism on the stability of the imino tautomers cannot be ignored. However, Dewar and co-workers [26] estimating the heat of atomization for the **AP** tautomers using the semi-empirical SCF MO method with π-approximation considered only three isomers (**AP16**, **IP13** and **IP15**) without any information, which isomers (**a** or **b**) for the imino tautomers have been selected. On the other hand, De Rosa and co-workers [27] took the geometrical isomerism for the imino isomers into account and applied higher levels of theory {HF, MP2, QCSID, and QCSID(T)} but chose the same three tautomers for investigations of tautomerism in **AP**. The other tautomer/rotamers have not been considered in their article. Our work completes the limited current knowledge on the topic. It can be mentioned here that when rotation about single/double bonds is structurally limited in isolated molecules or in crystal adducts, additional stabilizing hydrogen–hydrogen interactions may be considered [50,51]. 

The loss of the labile proton at the N1, C2, C3, C4, or C5 atom in the amino tautomers of **AP** gives the mono-anionic tautomer **AP6^−^** (Figure 3) of the lowest Gibbs energy. The Δ*G* values for the other ten tautomers/rotamers of AP^−^ are close to or higher than 40 kJ mol^−1^ (Table 2). According to the Mezey limit [47,48], only two additional isomers, **AP3a^−^** and **AP3b^−^**, can significantly contribute to the isomeric mixture. Since their percentage contents are low (0.0202 and 0.00002%, respectively), they may not be detected in experiments carried out in vacuo or in non-polar solvents. The other eight isomers can be treated as exceptionally rare forms. They possess Δ*G*s higher than 40 kJ mol^−1^. They may be considered in mechanisms of particular reactions carried out in the presence of extremely strong bases. To our knowledge, there are no literature data on prototropy in mono-deprotonated 2-aminopyrrole. According to our DFT calculations, the tautomer **AP6^−^** is preferred as a mono-deprotonated form. If we look at the major tautomer **AP16** of neutral 2-aminopyrrole, the exo N1 atom can be regarded as the favored site of deprotonation. This indicates that 2-aminopyrrole belongs to the family of organic NH-acids.

DFT-calculations carried out for all thirteen isomers of mono-protonated form of 2-aminopyrrole (Figure 4) clearly show that the tautomer **AP156^+^** has the lowest Gibbs energy (Table 3), and only one additional tautomer, **AP126^+^**, contributes significantly to the composition of the tautomeric mixture of **AP^+^** (Δ*G* < 40 kJ mol^−1^). However, its percentage content (0.006%) seems to be too low for experimental detection, and only the major tautomer **AP156^+^** can be experimentally observed in vacuo or in non-polar solvents. Indeed, the presence of the CH_2_ group in the mono-cation of 2-aminopyrrole has been confirmed in the ^1^H and ^13^C NMR spectra recorded for **AP^+^** acetate salt in glacial acetic acid [27], and also for **AP^+^** tetraphenylborate salt in (CD_3_)_2_CO [31], though solvent effects may have a significant impact on the fraction of rare isomers. The other eleven tautomers/rotamers of **AP^+^** possess Δ*G*s higher than 40 kJ mol^−1^ in vacuo, but they may be considered in mechanisms of particular reactions carried out in the presence of extremely strong acids.

Using various quantum chemical methods {HF, MP2, MP4SDQ, QCISD, and QCISD(T)} and different basis sets, Fradera et al. [28] investigated three tautomers of mono-protonated 2-aminopyrrole (**AP166^+^**, **AP156^+^**, and **AP136^+^**). Our DFT results for these three tautomers are in good agreement with those computed at the highest levels of theory {MP4SDQ, QCISD, and QCISD(T)}. At each level of theory, the tautomer **AP156^+^** is favored for the mono-protonated form. Since the tautomer **AP16** is the major form for neutral 2-aminopyrrole, the C5 atom can be considered as the favored site of mono-protonation. This means that 2-aminopyrrole, although possessing two N atoms, belongs to the family of organic C-bases.

### 2.3. HOMED Indices Estimated for Neutral and Ionic Tautomers/Rotamers of 2-Aminopyrrole

The stability order of tautomeric forms and electron delocalization are somehow related [38,39]. This can be measured quantitatively by the structural descriptor HOMED (Harmonic Oscillator Model of Electron Delocalization) [52,53], which is a recent modification of the HOMA (Harmonic Oscillator Model of Aromaticity) index, proposed fifty years ago by Kruszewski and Krygowski [54,55,56]. The HOMED descriptor can be applied to any delocalized compound, σ-π, n-π, and π-π conjugated systems, as well as aromatic molecules [49,52,53,57], whereas the HOMA index is mainly a quantitative measure of aromaticity [54,55,56]. For HOMED estimation, Equation (2) can be used here. It is analogous to that proposed by Krygowski in 1993 for the reformulated HOMA index [56]. For HOMED, only the parameterization is different [52,53] from that of HOMA [56].

HOMED = 1 − {*α*(CC)Σ[*R*_o_(CC) − *R*_i_(CC)]^2^ + *α*(CN)Σ[*R*_o_(CN) − *R*_i_(CN)]^2^}/*n* (2)

In Equation (2), *α*(CC) and *α*(CN) are the normalization constants for the CC and CN bonds, as described in Refs. [52,53]; *R*_o_(CC) and *R*_o_(CN) are the optimum bond-lengths for the reference molecules (benzene and 1,3,5-triazine) computed at the same DFT-level of theory as that applied for the neutral and ionic **AP**-isomers; *R*_i_(CC) and *R*_i_(CN) are the calculated bond-lengths in the investigated structures; and *n* is the number of bonds taken into account in the HOMED estimation. The HOMED parameters used in this work are given in Table S3 (Supplementary Materials). 

According to the parameterization procedure, the HOMED index is close to unity for aromatic compounds [52,53], like the original [54,55] and reformulated HOMA descriptor [56]. The two geometry-based parameters (HOMED and HOMA) have a common anchor point in the scale for aromatic hydrocarbons: benzene [52,53,54,55,56]. For N-containing aromatic systems, the two indices are close to unity when *R*_i_(CC) and *R*_i_(CN) are close to the defined *R*_o_(CC) and *R*_o_(CN) for the corresponding aromatic reference molecules. In such cases, investigated molecules present a resonance stability analogous to that in benzene and its hetero-analogs. Note that in N-containing tautomeric systems, aromatic isomers frequently possess the lowest Gibbs energies, and they predominate in the tautomeric mixtures [25,39,52,53]. 

On the other hand, the HOMED index is equal to zero for the hypothetical reference molecules cyclohexa-1,3,5-triene (Kekulé structure of benzene) and its triaza derivative (Kekulé structure of 1,3,5-triazine), possessing the localized CC and CN single and double bonds, lengths of which are equal to those for ethane, ethene, methylamine, and methylimine, respectively, like in the original HOMA index [54,55]. The resonance stabilization for this kind of system (hypothetical Kekulé structure) can be neglected. In the case of tautomeric systems, isomers with HOMED close to zero usually possess considerably higher Gibbs energies than the aromatic structures. The same is true for structures with negative HOMED values. This takes place in particular cases, when the CC and/or CN single bonds are longer than those in ethane and/or methylamine, and/or the CC and/or CN double bonds are shorter than those in ethene and/or methylimine. The negative HOMED values indicate that electrons in the investigated structures are less delocalized than in the hypothetical reference molecules.

Using the above-mentioned anchor points in the HOMED parameterization procedure (analogous aromatic structures, benzene and 1,3,5-triazine, for HOMED = 1, and their analogous hypothetical Kekulé structures for HOMED = 0), the HOMED index could appropriately measure any type of electron delocalization in σ-π, n-π, and π-π conjugated molecules. Meanwhile, the anchor points for defining zero in the reformulated HOMA procedure are based on differently conjugated reference molecules, i.e., moderately π-π conjugated 1,3-butadiene for CC bonds, and very weakly delocalized methylamine and methylimine for CN bonds [56]. These different measures of the CC and CN bonds have led to some discrepancies for some conjugated systems with HOMED < 0.9, particularly for rare CH tautomers of tautomeric hetero-compounds [49,53]. For this reason, we applied here the HOMED descriptor to quantitatively describe bond length alternation in **AP** isomers. For more details on parameterization of the HOMED index, see Refs. [52,53]. 

Table 4 summarizes the HOMED values calculated for all DFT structures of neutral (**AP**), mono-deprotonated (**AP^−^**), and mono-protonated (**AP^+^**) prototropic isomers of 2-aminopyrrole. HOMED5 corresponds to the pyrrole ring containing five bonds between heavy atoms, and HOMED6 refers to the entire molecule with six bonds, including the C2N6 bond. Among possible prototropic isomers of neutral 2-aminopyrrole, only one NHNH tautomer (**AP16**) of the lowest Gibbs energy displays an aromatic character for the ring (HOMED5 0.924). The aromaticity of the pyrrole ring in 2-aminopyrrole has also been confirmed by the magnetic criterion NICS (Nucleus-Independent Chemical Shifts). Elguero and co-workers [58], investigating series of aminoazoles, found a strongly negative NICS value (−14.7) for the ring in 2-aminopyrrrole, like for other aromatic azoles. **AP16** contains six labile electrons (two n-electrons of the endo N1 atom and four π-electrons of the endo C2–C5 atoms) well delocalized in the ring. The other NHCH isomers possess one C-sp^3^ and four labile electrons in the ring. Consequently, they lose the ring aromaticity, their π-electrons being considerably less delocalized than in **AP16**. Their HOMED5 indices are not higher than 0.5. Two configurational isomers of the imino tautomer (**IP34a** and **IP34b**), containing the labile protons at two C-sp^3^ (C3 and C4) and one pair of labile electrons in the ring, have the lowest HOMED5 values (close to zero). When the exo N6 atom is included in the structural-descriptor estimations, the variations of the HOMED6 values are analogous to those of HOMED5. The highest HOMED6 value is for the tautomer **AP16** (0.825), and the lowest ones are for **IP34a** and **IP34b** (close to zero). For other isomers, the HOMED6 values are not higher than 0.5.

Perusal of the HOMED indices calculated for all eleven mono-anionic isomers of 2-aminopyrrole indicates that the favored NH isomer **AP6^−^** has the most delocalized labile electrons. Its HOMED5 value is close to unity (0.958) and corresponds to an aromatic system. The mono-deprotonation at N1 increases electron delocalization in the ring in comparison to that in the neutral tautomer **A16** (0.924). Together with electron delocalization, the negative charge is also well delocalized in the ring. On the other hand, the exo NH_2_ group in **AP6^−^** participates to a lesser extent in electron and charge delocalization despite its electron-donor effect. The C2N6 bond length (1.4438 Å) in **AP6^−^** is closer to the single CN bond in methylamine computed at the same DFT level (1.4658 Å) than that in **AP16** (1.4169 Å). Consequently, the value of HOMED6 for **AP6^−^** is considerably lower (0.777) than that for **AP16** (0.825). The other NH isomers, **AP1a^−^** and **AP1b^−^**, seem to be moderately delocalized. Their HOMED5 and HOMED6 values are close to 0.8. The exo group participates in electron and charge delocalization. Note that the HOMED values are higher for **AP1b^−^** than **AP1a^−^**, indicating important effects of intramolecular interactions on electron delocalization. The favorable interaction between N6H and the n-electron pair of N1 in **AP1b^−^** increases electron delocalization in the system and the HOMED values in comparison to **AP1a^−^**, in which the unfavorable interaction (repulsion) between N1H and N6H takes place. These effects of intramolecular interactions between the functional groups influence also the HOMED values in the neutral prototropic isomers, but they are weaker than those in mono-anionic isomers. The HOMED indices for all CH isomers are lower than 0.4 because of the C-sp^3^ presence in the five-membered ring. The isomer **AP3a^−^** shows the highest HOMED values (close to 0.4), for which both electron and charge can be delocalized, and, additionally, the n-electrons of N1 can favorably interact with N6H. The negative HOMED values are calculated for isomers **AP2a^−^** and **AP2b^−^**, for which the negative charge cannot be delocalized with labile electrons. 

Since 2-aminopyrrole seems to be favorably mono-protonated at the endo C5 atom, the tautomer **AP156^+^** of the lowest Gibbs energy is moderately delocalized. Its HOMED5 (0.468) and HOMED6 (0.606) values are lower than those (0.922 and 0.669, respectively) of **AP166^+^**, possessing higher Gibbs energy, but still showing some aromaticity of the ring after mono-protonation at N6. In **AP156^+^**, the exo N6 atom with its n-electron pair can participate in delocalization of the labile electrons and charge (like in less stable **AP136^+^** and **AP146^+^**), while in **AP166^+^** there is no such possibility. Consequently, the C2N6 bond length in **AP156^+^** (1.3247 Å) is close to the CN bond length in aromatic 1,3,5-triazine (1.3342 Å), while that in **AP166^+^** (1.4641 Å) is close to the CN single bond in methylamine (1.4658 Å). These trends explain the differences between the HOMED5 and HOMED6 values for the two tautomers: HOMED5 < HOMED6 for **AP156^+^** and HOMED5 > HOMED6 for **AP166^+^**. The lack of participation of N6 in electron delocalization takes also place for other tautomers of high Gibbs energies protonated at this atom, such as **AP266^+^**, **AP366^+^**, **AP466^+^**, and **AP566^+^**, for which HOMED5 is between 0.3 and 0.5, while HOMED6 is lower than 0.1. The mono-protonation of **AP** at N1 is unlikely because it completely destroys electron delocalization and aromaticity of the ring. The two HOMED indices are close to zero for **AP116^+^**. The same is true for **AP126^+^**, **AP134a^+^**, and **AP134b^+^**. 

### 2.4. Microscopic Gas-Phase Proton Acidity/Basicity Parameters for Tautomers/Rotamers of **AP**

On the basis of the thermochemical data calculated at the DFT level for individual tautomers/rotamers of neutral, mono-deprotonated, and mono-protonated 2-aminopyrrole (Table S2 in Supplementary Material), we can estimate the so-called microscopic (partial) acidity/basicity parameters for particular acid/base sites. For example, for the favored neutral tautomer **AP16** (Figure 2), we can calculate the acidity parameters for the acid NH groups (N1H and N6H) containing the tautomeric (labile) protons and the basicity parameters for each heavy atom (N1, C2, C3, C4, C5, and N6).

Generally, in the gas phase, the acid/base strength can be measured in two scales [20,21,22]. In one of them, deprotonation enthalpy (DPE), frequently applied for neutral acids (AH), and proton affinity (PA), usually employed for neutral bases (B), correspond to the enthalpy change of the deprotonation/protonation Equilibrium (3). In the other one, gas-phase acidity (GA) and gas-phase basicity (GB) refer to the Gibbs energy change of the same Equilibrium (3). The commonly used terms and abbreviations for gas-phase proton acidity/basicity parameters (DPE or PA, and GA or GB) have historical grounds and are adopted here.

AH (or BH^+^) ⇄ A^−^ (or B) + H^+^ (3)

The microscopic acidity (DPE_micro_ and GA_micro_) parameters for acid groups and the microscopic basicity (PA_micro_ and GB_micro_) parameters for the base sites can be calculated using Equations (4) and (5), respectively. In these equations, *H*_i_ and *G*_i_ refer to the enthalpy and Gibbs energy, respectively, calculated at 298.15 K for the corresponding tautomers playing the base or acid role; *H*(H^+^) = 6.2 kJ mol^−1^, and *G*(H^+^) = −26.3 kJ mol^−1^ at 298.15 K [59,60]. The Equations (4) and (5) are analogous to those for compounds being free of prototropy [20,21,22]. Note that a stronger base has higher PA and GB values, and a stronger acid has lower DPE and GA values.

DPE_micro_(AH or BH^+^) = *H*_i_(A^−^ or B) + *H*(H^+^) − *H*_i_(AH or BH^+^) = PA_micro_(A^−^ or B) (4)

GA_micro_(AH or BH^+^) = *G*_i_(A^−^ or B) + *G*(H^+^) − *G*_i_(AH or BH^+^) = GB_micro_(A^−^ or B) (5)

**Figure 5:**
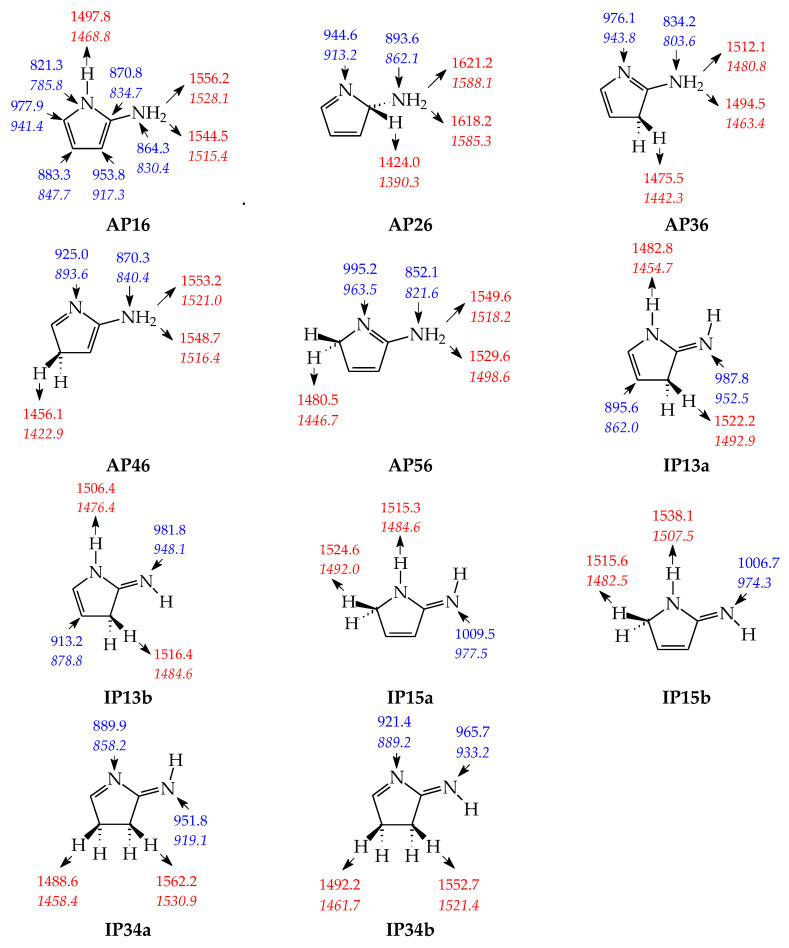
Microscopic gas-phase proton acidity/basicity parameters calculated at the DFT level for particular acid and base sites in isomers of neutral 2-aminopyrrole. DPE/GA for acid sites and PA/GB for base sites are indicated in red and blue (normal/italic style), respectively.

The DFT-calculated microscopic acidity/basicity parameters for all eleven prototropic isomers of neutral 2-aminopyrrole are included in Figure 5. First perusal of their values shows that change of positions of labile protons affect both the acidity of NH and CH groups, as well as the basicity of heavy atoms. For individual isomers, the DPE/GA and PA/GB values indicate also which base site displays the stronger basicity and can be considered as the favored site of protonation and which acid site exhibits the stronger acidity and can be treated as the preferred site of deprotonation. 

In the case of **AP16**, the PA/GB order for heavy atoms is as follows: C5 > C3 > C4 > C2 > N6 > N1. The C5 atom seems to be the favored site of protonation and the N1 atom the last one. However, the N1H group is more acidic than N6H and seems to be preferentially deprotonated. Quite a different situation takes place for the amino CH tautomers **AP26**, **AP36**, **AP46**, and **AP56**. The DPE/GA values for N6H are higher than those for C2H, C3H, C4H, and C5H, respectively, and thus the corresponding CH group (possessing the labile proton) is more easily deprotonated. Among N atoms, the imino N1 atom displays higher basicity parameters and is the most favorably protonated for the amino tautomers. For both isomers (**a** and **b**) of the imino tautomers **IP13**, **IP15**, and **IP34**, the situation is different, and N6 can be preferentially protonated. Concerning mono-deprotonation, N1H appears to be favorably deprotonated in **IP13a**, **IP13b**, and **IP15a**, while it is C5H in **IP15b** and C4H in **IP34a** and **IP34b**. Interestingly, the imino N6 atom in **IP15a** and **IP15b** exhibits very high PA values (>1000 kJ mol^−1^), indicating that derivatives of **IP15a** and **IP15b**, being free of prototropy, can be good candidates for superbases (see Section 2.6).

### 2.5. Thermochemistry of Acid/Base Equilibria in Vacuo for **AP**

**Scheme 1:**
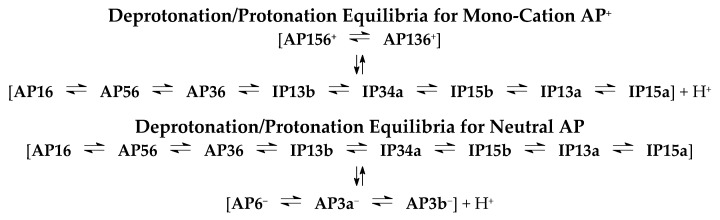
Tautomeric and acid/base equilibria possible for 2-aminopyrrole during gas-phase proton acidity/basicity measurements according to the equilibrium method. Participation of reference molecules is not shown. Note that in FT-ICR (as well as in other mass spectrometry techniques), the equilibria involving positive and negative ions are studied separately.

When the equilibrium method is applied for gas-phase proton acidity/basicity measurements, a mixture of isomeric molecules of tautomeric compound can be present in the FT-ICR (Fourier Transform Ion Cyclotron Resonance) mass spectrometer apparatus. In this technique, positive and negative ions are studied separately. After ionization, in positive or negative mode, the ionic forms (mono-cation **AP^+^** or mono-anion **AP**^−^) can be produced. Interacting with the corresponding reference molecules (base B or acid AH) introduced into the FT-ICR cell, the acid/base equilibria can be established and the acidity/basicity parameters measured. We can write the following general tautomeric and acid/base equilibria for 2-aminopyrrole, considering the Mezey limit of isomer significance [47,48]. In Scheme 1, the tautomeric mixtures of mono-cationic, neutral, and mono-anionic forms contain two, eight, and three isomers, respectively (see their relative Gibbs energies and percentage contents in Table 1, Table 2 and Table 3, respectively). All of them can be considered for quantum chemical estimation of the macroscopic acidity/basicity parameters.

In the case of the mono-deprotonation of the neutral tautomeric mixture (**AP**) to the mono-anionic tautomeric mixture (**AP^−^**), Equation (6) can be used for estimation of the macroscopic gas-phase acidity parameter, GA_macro_. In the case of the mono-deprotonation reaction of the mono-cationic tautomeric mixture (**AP^+^**) to the neutral tautomeric mixture (**AP**), Equation (7) can be employed for estimation of the macroscopic gas-phase basicity parameter, GB_macro_. As could be expected, the GA_macro_ and GB_macro_ values (1468.8 and 941.4 kJ mol^−1^, respectively) calculated for the corresponding reactions given in Scheme 1 are equal to the GA_micro_ and GB_micro_ of N1H and C5 in **AP16**, respectively (Figure 5). The reason is that the percentage contents of **AP6^−^**, **AP16**, and **AP56^+^** are very high (>99.9%) in comparison to those of other isomers, for which their contribution in the tautomeric mixtures are exceptionally low. For the same reason, the DPE_macro_ and PA_macro_ values are estimated in an analogous way as for GA_macro_ and GB_macro_, but in the enthalpy scale (1497.8 and 977.9 kJ mol^−1^, respectively) are equal to the DPE_micro_ and PA_micro_ of N1H and C5 in **AP16**, respectively (Figure 5). From a statistical point of view, this indicates that at the DFT level, only the favored isomers can be considered for gaseous acid/base equilibria of 2-aminopyrrole.

GA_macro_(**AP**) = Σ_1_^n^*x*_i_*G*(**AP_i_^−^**) + *G*(H^+^) − Σ_1_^n^*x*_i_*G*(**AP_i_**) (6)

GB_macro_ (**AP**) = Σ_1_^n^*x*_i_*G*(**AP_i_**) + *G*(H^+^) − Σ_1_^n^*x*_i_*G*(**AP_i_**^+^) (7)

To our knowledge, there are no experimental nor theoretical gas-phase proton acidity/basicity data for 2-aminopyrrole reported in the literature. However, we can compare the DPE, GA, PA, and GB calculated for **AP16** with the literature experimental data for unsubstituted pyrrole [19,20,21,22]. **AP** seems to possess almost the same acid strength of the N1H group, but it seems to be a considerably stronger C-base (by ca. 100 kJ mol^−1^) than its parent system. This observation indicates that the strong electron-donor effect of the exo NH_2_ group could be responsible for the enhancement of basicity of C5 in **AP**. 

### 2.6. Importance of CH Tautomers in the Design of Superbasic Imines

As signaled above, some tautomers of 2-aminopyrrole possessing one labile proton at the C atom display exceptionally high PAs of the imino N atom (close to or higher than 1000 kJ mol^−1^). This takes place for the imino N1 atom in the amino tautomers **AP36** and **AP56**, and also for the imino N6 atom in the imino isomers **IP13a**, **IP13b**, **IP15a**, and **IP15b**. Since these isomers are rare forms, their acidity/basicity parameters can be difficult or even impossible to measure. Nevertheless, certain alkyl derivatives, being free of prototropy, may be synthesized and their gas-phase basicity parameters measured. 

**Figure 6:**
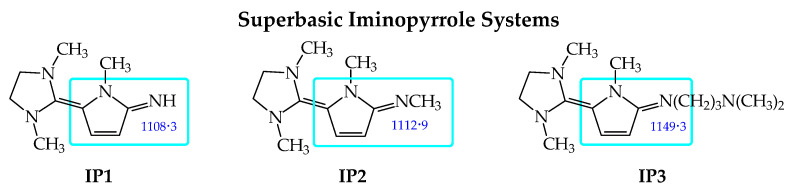
Push–pull superbases in which the iminopyrrole part of the CH tautomer (**IP15**) of 2-aminopyrrole is framed in turquoise. Their Pas (in kJ mol^−1^), calculated for the imino N atom as the protonation site using the Hartree–Fock model and scaled for organic N-bases by Maksić and co-workers [23], are indicated in blue.

Among the molecules studied by the Maksić group [23], we can select derivatives, for which superbasicity takes its origin from the imino CH tautomer **IP15** of 2-aminopyrrole. Three iminopyrrole systems (**IP1**–**IP3**, given in Figure 6) are free of tautomeric conversions, and their basicity parameters (PAs > 1000 kJ mol^−1^) show that they can be classified as superbases. However, the effect of geometrical isomerism about the C=N double bond on basicity parameter has not been discussed, and the PA values, estimated using the Hartree–Fock model scaled for organic N-bases, contain some uncertainty. Difference between PAs of **IP1** and **IP2** are attributed to the polarizability effect of the −CH_3_ group present at the imino N atom (site of protonation), whereas that between the PAs of **IP1** and **IP3** may be a consequence of both the polarizability and field/inductive effects of the –(CH_2_)_3_N(CH_3_)_2_ substituent linked to the site of protonation as well as the chelation effect of the proton by two basic sites, the imino N atom in the iminopyrrole part and the amino N atom in a “scorpio” conformation of the substituent. Note that a diradical character discussed recently for N-heterocyclic quinodimethane [61] has not been documented for **IP1**–**IP3** in Ref. [23]. No information on the wave-function stabilities for these systems has been given in that work, nor in the review article [24]. The closed-shell (singlet) structures for the base and mono-protonated forms have been studied, and the PA values for the imino N-atom predicted. It would be interesting to study the possible open-shell (diradical) structures for various push–pull iminopyrroles (being free of prototropy), and to characterize their diradical character by different descriptors based on the structure, reactivity, orbital overlap, etc. Such an investigation and characterization is out of the scope of the present work.

**Figure 7:**
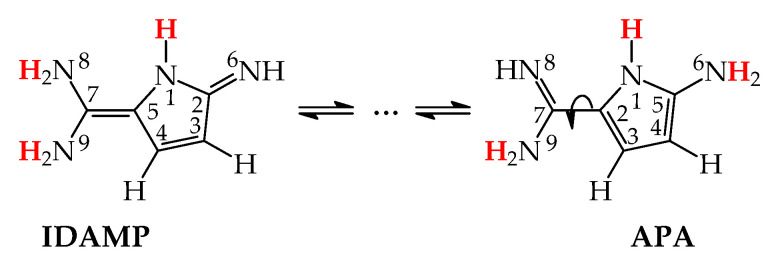
Structure of 2-imino-5-diaminomethylidene-3-pyrroline (**IDAMP**, proposed as a strong imino N-base by Maksić in Ref. [23]) and 5-aminopyrrole-2-amidine (**APA**, expected favored form of **IDAMP**). Labile (tautomeric) protons are indicated in bold red. The numbering of heavy atoms is displayed in **IDAMP** and **APA** formulae.

Unfortunately, the other iminopyrrole systems with the **IP15** fragment, proposed by the Masić group in Ref. [23] as candidates for new superbases, possess labile (tautomeric) protons, and thus, they can tautomerize to more stable amino forms. For example, 2-imino-5-diaminomethylidene-3-pyrroline (IDAMP, shown in Figure 7) contains three tautomeric protons at amino N atoms and nine conjugated tautomeric sites (all heavy atoms). Like in 2-aminopyrrole, heavy atoms of exo groups can participate in the tautomeric conversions together with heavy atoms of the pyrrole ring. For example, the exo groups, imino (=N6H) at the 2-position and diaminomethylidene {=C7(NH_2_)_2_} at the 5-position, can convert during tautomerization to more favorable tautomeric groups, amino (−NH_2_) and carboximidamido {amidine, −C(=NH)NH_2_} substituents, leading to the expected 5-aminopyrrole-2-amidine (**APA**, IUPAC name: 5-amino-1H-pyrrole-2-carboximidamide), possessing the aromatic pyrrole ring. This exciting prototropy phenomenon and its effect on the favored site of protonation and gas-phase basicity parameters will be examined in the future. 

## 3. Computational Details

For all quantum chemical calculations, the Gaussian 03 programs were used [62]. Geometries of all neutral and ionic isomers of investigated pyrrole derivatives were optimized without symmetry constraint in their ground states in vacuo at the DFT(B3LYP)/6-311+G(d,p) level [41,42,43,44], as previously described in Refs. [40,49,52,53,57,63]. Their atom coordinates and DFT-calculated electronic energies are summarized in Table S1 (Supplementary Materials). Vibrational frequencies and thermochemical parameters such as enthalpy (*H*), entropy (*S*), and Gibbs energy (*G*) were calculated at 298.15 K using the same method as that employed for geometry optimization. The DFT-calculated thermochemical data, such as *H*, *G*, and *S*, are listed in Table S2 (Supplementary Materials). The parameters of Equation (2) applied in the HOMED estimations and taken from Refs. [52,53] are included in Table S3 (Supplementary Materials).

## 4. Conclusions

Application of quantum chemical methods to the tautomeric 2-aminopyrrole (**AP**) system opened the possibility to study the intramolecular proton-transfer equilibria (prototropy). Simultaneously, the intermolecular (acid/base) proton-transfer reactions could be examined. In this way, the thermochemistry of the corresponding proton-transfer processes could be determined in vacuo. We analyzed the properties of all possible tautomers for neutral as well as for ionic (mono-deprotonated and mono-protonated) forms, and estimated their amounts in the tautomeric mixtures. We also determined acidity/basicity parameters at the micro- and macro-scale. Using **AP** as a simple example of a tautomeric derivative, we could explain the origin of the strong basicity of some iminopyrroles proposed in the literature [23,24] being free of prototropy. Factors that dictate the strong basicity of N-bases have been recently discussed [64]. Here, we highlighted the role of CH tautomers in the design of strong Brønsted imino N-bases. We additionally reported that the iminopyrrole systems containing tautomeric protons most probably tautomerize to more favorable tautomeric aminopyrrole derivatives, like **AP**, displaying considerably lower basicity parameters than iminopyrroles. The nature of the site of protonation and of substituent effects explain this striking difference.

## Figures and Tables

**Table 1:** Relative enthalpies, Gibbs energies, entropy terms (Δ*H*, Δ*G*, and *T*Δ*S* in kJ mol**^−^**^1^ at 298.15 K), and mole fractions (*x*_i_ in %) calculated at the DFT level for isomers of neutral 2-aminopyrrole (structures given in Figure 2).

Neutral Isomer	Δ*H*	*−T*Δ*S*	Δ*G*	*x*_i_
**AP16**	0.0	0.0	0.0	99.9838
**AP56**	17.4	4.7	22.1	0.0135
**AP36**	22.4	4.1	26.4	0.0023
**IP13b**	28.1	2.7	30.8	0.0004
**IP34a**	28.3	3.1	31.4	0.0003
**IP15b**	28.8	4.0	32.8	0.0002
**IP13a**	34.0	1.1	35.2	<0.0001
**IP15a**	31.7	4.4	36.0	<0.0001
**IP34b**	42.2	3.3	45.5	<0.0001
**AP46**	41.7	4.2	45.9	<0.0001
**AP26**	73.8	4.5	78.4	<0.0001

**Table 2:** Relative enthalpies, Gibbs energies, entropy terms (Δ*H*, Δ*G*, and *T*Δ*S* in kJ mol**^−^**^1^ at 298.15 K), and mole fractions (*x*_i_ in %) estimated at the DFT level for mono-deprotonated AP^−^ isomers (structures displayed in Figure 3).

Anionic Isomer	Δ*H*	*−T*Δ*S*	Δ*G*	*x*_i_
**AP6^−^**	0.0	0.0	0.0	99.9798
**AP3a^−^**	19.0	2.1	21.1	0.0202
**AP3b^−^**	36.6	1.8	38.4	<0.0001
**AP1b^−^**	46.6	0.0	46.6	<0.0001
**AP5a^−^**	49.1	2.8	51.9	<0.0001
**AP1a^−^**	58.4	0.9	59.3	<0.0001
**AP5b^−^**	69.1	2.4	71.6	<0.0001
**AP4a^−^**	92.6	0.9	93.5	<0.0001
**AP4b^−^**	97.1	1.1	98.2	<0.0001
**AP2a^−^**	194.3	0.7	195.0	<0.0001
**AP2b^−^**	197.2	0.6	197.8	<0.0001

**Table 3:** Relative enthalpies, Gibbs energies, entropy terms (Δ*H*, Δ*G*, and *T*Δ*S* in kJ mol**^−^**^1^ at 298.15 K), and mole fractions (*x*_i_ in %) estimated at the DFT level for mono-protonated **AP^+^** isomers (structures presented in Figure 4).

Cationic Isomer	Δ*H*	*−T*Δ*S*	Δ*G*	*x*_i_
**AP156^+^**	0.0	0.0	0.0	99.9940
**AP136^+^**	24.1	0.0	24.1	0.0060
**AP346^+^**	54.3	−0.6	53.8	<0.0001
**AP146^+^**	94.6	−0.8	93.7	<0.0001
**AP134b^+^**	98.7	−0.9	97.8	<0.0001
**AP126^+^**	107.1	−0.4	106.7	<0.0001
**AP166^+^**	113.5	−2.5	111.0	<0.0001
**AP134a^+^**	116.3	−1.6	114.6	<0.0001
**AP566^+^**	143.1	−1.1	142.0	<0.0001
**AP466^+^**	149.3	−2.3	147.0	<0.0001
**AP116^+^**	156.6	−1.0	155.6	<0.0001
**AP266^+^**	158.2	−0.4	157.7	<0.0001
**AP366^+^**	165.9	−1.6	164.3	<0.0001

**Table 4:** HOMED indices calculated for DFT structures of all possible prototropic isomers of neutral and ionic 2-aminopyrrole. HOMED5 and HOMED6 refer to the five-membered ring and to the entire molecule, respectively.

Isomer	HOMED5	HOMED6	Isomer	HOMED5	HOMED6
**AP16**	0.924	0.825	**AP4b^−^**	0.275	0.321
**AP26**	0.231	0.077	**AP5a^−^**	0.329	0.371
**AP36**	0.388	0.411	**AP5b^−^**	0.305	0.349
**AP46**	0.434	0.437	**AP6^−^**	0.958	0.777
**AP56**	0.381	0.393	**AP116^+^**	−0.050	0.005
**IP13a**	0.314	0.304	**AP126^+^**	0.087	0.074
**IP13b**	0.306	0.298	**AP136^+^**	0.382	0.419
**IP15a**	0.334	0.333	**AP146^+^**	0.487	0.502
**IP15b**	0.360	0.358	**AP156^+^**	0.468	0.606
**IP34a**	−0.029	−0.036	**AP166^+^**	0.922	0.669
**IP34b**	−0.056	−0.057	**AP266^+^**	0.363	−0.130
**AP1a^−^**	0.755	0.772	**AP366^+^**	0.385	0.082
**AP1b^−^**	0.816	0.828	**AP466^+^**	0.492	0.072
**AP2a^−^**	−0.182	−0.164	**AP566^+^**	0.403	0.072
**AP2b^−^**	−0.271	−0.283	**AP134a^+^**	−0.127	−0.179
**AP3a^−^**	0.362	0.390	**AP134b^+^**	−0.063	−0.108
**AP3b^−^**	0.305	0.340	**AP346^+^**	0.280	0.307
**AP4a^−^**	0.220	0.269			

## Data Availability

The original contributions presented in this study are included in the article/Supplementary Material. Further inquiries can be directed to the corresponding author.

## Supplementary Materials

The following information can be downloaded at https://www.mdpi.com/article/10.3390/molecules30102112/s1. Table S1. DFT-calculated atom coordinates and electronic energies for neutral and ionic isomers of 2-aminopyrrole. Table S2. DFT-calculated enthalpies, Gibbs energies, and entropies for neutral and ionic isomers of 2-aminopyrrole. Table S3. Parameters used for HOMED estimation. Scheme S1. Resonance structures for 2-aminopyrrole showing acid and base sites for intramolecular proton-transfers (prototropy). Scheme S2. Resonance structures for the imino tautomers explaining isomerism of exo imino group.
