# Supplementary material for: Quantum Chemical Studies on the Prototropic and Acid/Base Equilibria for 2-Aminopyrrole in Vacuo—Role of CH Tautomers in the Design of Strong Brønsted Imino N-Bases
Source: Molecules. 2025 May 9;30(10):2112. doi: 10.3390/molecules30102112 (PMC12113883; doi:10.3390/molecules30102112)
Supplement: Supplementary file 1 [file molecules-30-02112-s001.zip › molecules-3620990-supplementary.pdf]

Supplementary Materials

Quantum Chemical Studies on the Prototropic and Acid/Base Equilibria for 2-Aminopyrrole in Vacuo—Role of CH Tautomers in the Design of Strong Brønsted Imino N-Bases

Ewa Daniela Raczyńska,[1] Pierre-Charles Maria,[2] and Jean-François Gal[2]

[1] Department of Chemistry, Warsaw University of Life Sciences (SGGW), ul. Nowoursynowska 159c, 02-776 Warsaw, Poland
[2] Institut de Chimie de Nice, UMR 7272, Université Côte d'Azur, Parc Valrose, 06108 Nice, France

**Contents**	**Page**
DFT-calculated atom coordinates and electronic energies for neutral and ionic isomers of 2-aminopyrrole (Table S1)	S2
DFT-calculated enthalpies, Gibbs energies, and entropies for neutral and ionic isomers of 2-aminopyrrole (Table S2)	S8
Parameters used for HOMED estimation (Table S3)	S9
Resonance structures for 2-aminopyrrole (Scheme S1) showing acid and base sites for intramolecular proton-transfers (prototropy)	S9
Resonance structures for imino tautomers (Scheme S2) explaining isomerism of the exo imino group	S10

Table S1. DFT-calculated atom coordinates (in Angstroms) and electronic energies (*E* in Hartree) for neutral and ionic isomers of 2-aminopyrrole.

AP16

E = –265.602803

N	-1.286968	-0.844153	-0.142504
C	0.020421	-1.261904	-0.100941
C	0.805636	-0.147596	0.099124
C	-0.062315	0.979070	0.182066
C	-1.355433	0.519744	0.029174
N	-2.628176	1.142409	0.017155
H	-2.102015	-1.420567	-0.277040
H	0.282947	-2.300790	-0.215716
H	1.881771	-0.141616	0.177666
H	0.222923	2.008713	0.335908
H	-2.847596	1.598942	0.894928
H	-2.724836	1.817768	-0.732180

AP26

E = –265.574143

N	-1.264565	-0.969945	-0.215202
C	0.004950	-1.126956	-0.324325
C	0.800866	-0.067010	0.328049
C	-0.079226	0.786784	0.869444
C	-1.465017	0.248950	0.606277
N	-2.386985	1.226583	0.061851
H	0.427655	-1.970508	-0.864789
H	1.880963	-0.010846	0.346283
H	0.118817	1.692211	1.425724
H	-2.050693	1.559704	-0.837145
H	-3.290911	0.792872	-0.098584
H	-1.881604	-0.112488	1.558217

AP36

E = –265.593180

N	-1.196021	-0.750637	-0.252258
C	0.144596	-1.178980	-0.084555
C	0.983347	-0.204045	0.305302
C	0.157071	1.049802	0.434899
C	-1.204312	0.511309	0.038873
N	-2.339051	1.273307	0.054217
H	0.381921	-2.216149	-0.279967
H	2.044644	-0.275941	0.488670
H	0.139017	1.455506	1.452688
H	-2.255940	2.273653	-0.021694
H	-3.158399	0.862353	-0.368334
H	0.486557	1.856812	-0.230481

AP46

E = –265.585395

N	-1.161869	-0.818209	-0.251042
C	0.037297	-1.239062	-0.064930
C	0.962939	-0.189983	0.483370
C	0.029743	0.980567	0.578126
C	-1.179160	0.551955	0.146365
N	-2.405728	1.182776	0.078107
H	0.320951	-2.261214	-0.295745
H	1.818014	-0.033413	-0.189071
H	0.290311	1.963466	0.939986
H	-2.396069	2.190141	0.041033
H	-3.058210	0.753357	-0.561291
H	1.385310	-0.508362	1.447041

AP56

E = –265.595294

N	-1.247280	-0.771839	-0.430361
C	0.129721	-1.217255	-0.261965
C	0.883057	-0.055044	0.325260
C	0.010517	0.949844	0.485828
C	-1.290841	0.444996	0.002270
N	-2.447985	1.188961	0.069238
H	0.170710	-2.098191	0.392934
H	1.936307	-0.055121	0.573425
H	0.197025	1.934960	0.893485
H	-2.356860	2.191859	0.029462
H	-3.228390	0.814194	-0.450563
H	0.548108	-1.533604	-1.226643

IP13a

E = –265.588697

N	-1.255141	-0.809574	-0.250151
C	0.117460	-1.107987	-0.246634
C	0.829256	-0.093929	0.255727
C	-0.121656	1.015592	0.640098
C	-1.496822	0.460986	0.271083
N	-2.598719	1.081032	0.431337
H	0.456174	-2.062384	-0.623069
H	1.901442	-0.067941	0.369359
H	-0.094083	1.259867	1.706583
H	-3.399061	0.529951	0.118725
H	0.059667	1.948974	0.098135
H	-1.975907	-1.427967	-0.583513

IP13b

E = −265.591146

N	-1.235021	-0.728179	-0.437019
C	0.128160	-1.038449	-0.410425
C	0.833937	-0.078978	0.200309
C	-0.123179	1.004715	0.643914
C	-1.492157	0.488171	0.177102
N	-2.666889	0.974885	0.273727
H	-0.127316	1.154579	1.729013
H	0.474341	-1.960668	-0.853870
H	0.088646	1.977145	0.186370
H	1.901461	-0.073028	0.353038
H	-2.652150	1.871338	0.754793
H	-1.965743	-1.293431	-0.837183

IP15a

E = −265.590090

N	-1.227204	-0.897789	-0.219204
C	0.190973	-1.212536	-0.337985
C	0.835487	0.038726	0.197311
C	-0.094062	0.953031	0.485161
C	-1.437573	0.423481	0.170979
N	-2.522665	1.095520	0.271806
H	0.463224	-2.098350	0.250072
H	1.905717	0.135404	0.320460
H	-3.338433	0.542919	0.005154
H	0.048817	1.948090	0.879656
H	-1.918970	-1.391119	-0.760156
H	0.490640	-1.403528	-1.378373

IP15b

E = −265.591228

N	-1.179549	-0.764180	-0.541137
C	0.218246	-1.147703	-0.476795
C	0.820347	-0.004061	0.295765
C	-0.133432	0.850976	0.673376
C	-1.457277	0.363774	0.209725
N	-2.643158	0.805810	0.408988
H	-0.008328	1.761683	1.242685
H	0.667239	-1.246192	-1.474059
H	1.881684	0.074991	0.490164
H	-2.620088	1.650024	0.975614
H	-1.918774	-1.403541	-0.783246
H	0.370451	-2.103411	0.045879

IP34a

E = −265.591180

N	-1.214807	-0.780551	-0.448795
C	0.046190	-0.973969	-0.524862
C	0.935742	0.098814	0.058001
C	-0.098946	1.115784	0.572106
C	-1.435128	0.476391	0.208335
N	-2.570474	0.979538	0.450067
H	1.606526	0.501814	-0.707766
H	-3.303597	0.355377	0.106592
H	-0.043096	1.274914	1.650089
H	0.438282	-1.875755	-0.991534
H	-0.011370	2.093207	0.095171
H	1.574729	-0.315344	0.844725

IP34b

E = −265.585819

N	-1.198946	-0.771005	-0.457366
C	0.060298	-0.973504	-0.522963
C	0.958919	0.093466	0.060037
C	-0.071448	1.119288	0.560980
C	-1.420129	0.483814	0.188030
N	-2.597622	0.912957	0.373958
H	1.636959	0.489049	-0.702956
H	-0.015788	1.279811	1.639705
H	0.448656	-1.880374	-0.982198
H	0.033479	2.092510	0.076896
H	1.588011	-0.320678	0.854460
H	-2.578229	1.818816	0.845505

AP1a^{-}

E = −264.996630

N	-1.357361	-0.787136	0.140464
C	-0.031240	-1.263314	0.010837
C	0.777519	-0.147648	-0.007464
C	-0.034241	1.019490	0.024342
C	-1.399627	0.626045	0.056520
N	-2.508182	1.365326	0.003081
H	0.207344	-2.306722	0.154655
H	1.861039	-0.172070	-0.004562
H	0.297683	2.046730	-0.014986
H	-2.151068	-1.322380	-0.173391
H	-3.324456	0.758209	0.102565

AP1b^{-}

E = –265.001313

N	-1.301760	-0.690504	0.033671
C	0.003263	-1.203601	-0.020573
C	0.850975	-0.118871	0.037961
C	0.066312	1.071448	0.084111
C	-1.312696	0.708527	0.043468
N	-2.480605	1.356169	0.001458
H	0.197506	-2.264566	0.006017
H	1.932424	-0.178396	0.068179
H	0.433723	2.088530	0.108627
H	-2.290916	2.357093	0.002015
H	-2.144916	-1.201331	-0.166603

AP2a^{-}

E = –264.942283

N	-1.253319	-1.051401	-0.181000
C	0.016346	-1.158339	-0.304593
C	0.798521	-0.057008	0.296145
C	-0.102657	0.753353	0.880693
C	-1.519087	0.264842	0.607501
N	-2.402514	1.220132	0.053668
H	0.476856	-2.004315	-0.826327
H	1.876236	0.059373	0.228044
H	0.080090	1.680567	1.409437
H	-1.919701	1.636682	-0.751055
H	-1.947911	-0.122115	1.553198

AP2b^{-}

E = –264.942815

N	-1.272007	-1.015683	-0.186760
C	-0.001197	-1.155428	-0.282499
C	0.785187	-0.071094	0.333306
C	-0.103523	0.795705	0.845651
C	-1.508671	0.300908	0.603181
N	-2.359982	1.271490	0.067758
H	0.454852	-2.017446	-0.782326
H	1.868890	-0.006902	0.345088
H	0.093971	1.734356	1.347217
H	-3.287658	0.847240	-0.013700
H	-1.813030	-0.177654	1.593234

AP3a^{-}

E = –265.012093

N	-1.727784	-0.467275	-0.588202
C	-0.475694	-0.996023	-0.367638
C	0.488834	-0.128787	0.053184
C	-0.195069	1.216301	0.140813
C	-1.634402	0.866621	-0.301251
N	-2.566798	1.779077	-0.363356
H	1.524139	-0.349064	0.278158
H	-0.208687	1.651655	1.148397
H	-0.326452	-2.061720	-0.538844
H	0.225184	1.981273	-0.524877
H	-3.423242	1.314730	-0.676904

AP3b^{-}

E = –265.005047

N	-1.730337	-0.503490	-0.503078
C	-0.460604	-1.003819	-0.347163
C	0.512288	-0.123323	0.029479
C	-0.192268	1.205342	0.156681
C	-1.659548	0.825979	-0.218376
N	-2.669201	1.659415	-0.253670
H	1.561351	-0.325485	0.201466
H	-0.161136	1.633735	1.168431
H	-0.299862	-2.065787	-0.530492
H	-2.329617	2.587598	0.004121
H	0.183245	1.978755	-0.528120

AP4a^{-}

E = –264.982529

N	-1.644894	-0.470929	-0.471474
C	-0.506000	-0.991443	-0.222338
C	0.563987	-0.003129	0.170083
C	-0.210590	1.272801	0.099749
C	-1.527666	0.995731	-0.282474
N	-2.578350	1.772619	-0.480319
H	1.422640	-0.100848	-0.525502
H	-0.342945	-2.069538	-0.294507
H	-3.355744	1.172796	-0.752914
H	0.970312	-0.282259	1.163912
H	0.185181	2.255609	0.311104

AP4b$^-$

$E = -264.980857$

N	-1.631372	-0.456944	-0.449817
C	-0.493259	-0.987644	-0.221141
C	0.594791	-0.017927	0.173505
C	-0.174483	1.263349	0.128981
C	-1.502114	0.994184	-0.243635
N	-2.597573	1.709212	-0.439406
H	1.443919	-0.110978	-0.533899
H	-0.342064	-2.065756	-0.310896
H	1.009626	-0.312878	1.158923
H	0.233509	2.240882	0.349919
H	-2.362809	2.688940	-0.262384

AP5a$^-$

$E = -265.000348$

N	-1.842273	-0.619606	-0.194875
C	-0.505193	-1.175187	-0.152103
C	0.445905	-0.010850	0.045844
C	-0.300052	1.097063	0.107512
C	-1.740405	0.713314	-0.043981
N	-2.674092	1.651476	-0.014673
H	1.527166	-0.088618	0.120369
H	0.031711	2.120609	0.242768
H	-3.577415	1.185700	-0.133837
H	-0.251313	-1.728590	-1.078763
H	-0.384860	-1.915781	0.664259

AP5b$^-$

$E = -264.992325$

N	-1.837619	-0.621975	-0.204219
C	-0.501401	-1.167862	-0.148448
C	0.448367	-0.002999	0.046371
C	-0.304317	1.099943	0.094897
C	-1.754247	0.707911	-0.064755
N	-2.761925	1.573591	-0.060289
H	1.529726	-0.074973	0.128521
H	0.040390	2.122722	0.225986
H	-0.239045	-1.727747	-1.068968
H	-0.382898	-1.902038	0.674124
H	-2.371411	2.507718	0.065951

AP6$^-$

$E = -265.020144$

N	-1.371380	-0.721773	0.041240
C	-0.081183	-1.178932	-0.023145
C	0.839879	-0.127726	0.024610
C	0.060950	1.059337	0.146367
C	-1.268324	0.623204	0.145199
N	-2.449591	1.446242	0.254048
H	0.124488	-2.242082	-0.098274
H	1.921483	-0.202288	-0.009744
H	0.412342	2.078891	0.250026
H	-3.223709	0.821313	0.463768
H	-2.665736	1.874444	-0.643764

AP116$^+$

$E = -265.926300$

N	-0.960754	-0.050167	-1.092120
C	0.387867	-0.511902	-0.663369
C	0.619077	0.005444	0.546018
C	-0.501549	0.836493	0.973637
C	-1.453580	0.837104	0.024465
N	-2.751192	1.254548	-0.067648
H	-1.615014	-0.834305	-1.201977
H	-3.023875	1.822165	-0.862204
H	-3.162719	1.582771	0.796605
H	0.969457	-1.106145	-1.348275
H	1.523807	-0.147629	1.116459
H	-0.555115	1.377950	1.905810
H	-0.918771	0.440112	-1.994322

AP126$^+$

$E = -265.944630$

N	-1.197646	-0.995321	-0.190007
C	0.083156	-1.183313	-0.285358
C	0.810230	-0.095952	0.329912
C	-0.095037	0.770672	0.824934
C	-1.500651	0.280234	0.596070
N	-2.402963	1.201478	0.040229
H	0.503934	-2.059322	-0.767256
H	1.887139	-0.029481	0.364903
H	0.094771	1.698542	1.347087
H	-2.153555	1.612015	-0.848935
H	-3.385718	0.985013	0.121854
H	-1.891310	-0.086035	1.557077
H	-1.907151	-1.630389	-0.540219

AP136$^+$

$E = -265.976404$

N	-0.595819	0.315906	-1.069368
C	0.562761	-0.371044	-0.579187
C	0.636250	-0.225839	0.741421
C	-0.531008	0.606666	1.206625
C	-1.259090	0.895306	-0.078568
N	-2.367986	1.603400	-0.201987
H	-2.815853	1.766136	-1.093901
H	-2.807949	2.010529	0.610882
H	-1.183440	0.075759	1.909594
H	-0.859324	0.345380	-2.046805
H	1.197951	-0.889538	-1.279548
H	1.395694	-0.627823	1.392740
H	-0.225176	1.539041	1.695341

AP146$^+$

$E = -265.948502$

N	-0.528097	0.251830	-1.080424
C	0.619437	-0.218482	-0.697221
C	0.759139	0.013229	0.754379
C	-0.502887	0.730740	1.111725
C	-1.262788	0.856962	0.004133
N	-2.532874	1.295524	-0.260593
H	-2.658221	1.941682	-1.030401
H	-3.077586	1.555616	0.550265
H	1.676685	0.577594	0.976480
H	-0.746465	1.089379	2.098879
H	-0.894085	0.178924	-2.027302
H	1.305582	-0.706096	-1.377331
H	0.891921	-0.955122	1.263442

AP156$^+$

$E = -265.986082$

N	-0.675746	0.119067	-1.084138
C	0.567087	-0.480209	-0.595396
C	0.604723	-0.000867	0.818215
C	-0.468807	0.760595	1.082554
C	-1.273265	0.829208	-0.130237
N	-2.413464	1.489627	-0.266588
H	-2.936216	1.504120	-1.130873
H	-2.798431	2.004783	0.511346
H	1.402387	-0.253491	1.502825
H	-0.726579	1.250563	2.009895
H	-1.020956	-0.001232	-2.026164
H	1.427338	-0.130987	-1.177095
H	0.530328	-1.573057	-0.664124

AP166$^+$

$E = -265.944224$

N	-0.643459	0.123584	-1.111905
C	0.559870	-0.315003	-0.607175
C	0.634423	0.044685	0.717140
C	-0.563937	0.731828	1.051623
C	-1.312938	0.755339	-0.095936
N	-2.637991	1.342856	-0.302450
H	-2.645410	2.096872	-1.003076
H	-2.950218	1.759163	0.581828
H	1.457201	-0.159331	1.382625
H	-0.828849	1.148845	2.011731
H	-0.946332	-0.013156	-2.064974
H	1.257313	-0.844635	-1.235232
H	-3.352522	0.653143	-0.572998

AP266$^+$

$E = -265.926872$

N	-1.295493	-0.911840	-0.133403
C	-0.037451	-1.132386	-0.291750
C	0.828665	-0.093435	0.314870
C	0.016173	0.820263	0.864948
C	-1.382632	0.282976	0.687003
N	-2.342229	1.218306	-0.033127
H	0.315341	-1.997988	-0.842613
H	1.909635	-0.091670	0.301585
H	0.285814	1.703669	1.427511
H	-1.939553	1.514527	-0.928638
H	-3.208097	0.706170	-0.239667
H	-1.852057	0.036927	1.645447
H	-2.570386	2.053462	0.514092

AP366$^+$

$E = -265.922718$

N	-0.650814	0.385222	-1.063905
C	0.608251	-0.094727	-0.592559
C	0.707608	0.002851	0.744054
C	-0.572884	0.595859	1.270892
C	-1.286871	0.759902	-0.033593
N	-2.649213	1.310687	-0.241149
H	-2.829648	1.281597	-1.254301
H	-2.737575	2.284435	0.074225
H	1.543672	-0.283775	1.364585
H	-0.413337	1.544902	1.798167
H	1.321777	-0.469329	-1.309898
H	-3.377280	0.761416	0.231810
H	-1.093586	-0.073870	1.966943

AP466$^+$

E = –265.929154

N	-0.423332	0.604829	-1.069832
C	0.657655	0.034202	-0.650664
C	0.600697	-0.329650	0.805835
C	-0.771086	0.151822	1.184738
C	-1.256660	0.660808	0.048025
N	-2.568349	1.284492	-0.202449
H	-3.070953	0.772398	-0.939118
H	-2.445672	2.246918	-0.543353
H	1.397946	0.158625	1.381963
H	1.497916	-0.146723	-1.310733
H	-3.154195	1.307552	0.637333
H	-1.223066	0.083014	2.163247
H	0.732749	-1.408217	0.964559

AP566$^+$

E = –265.931643

N	-0.652556	0.262577	-1.119536
C	0.633864	-0.242369	-0.639329
C	0.628868	-0.059281	0.847687
C	-0.548150	0.499125	1.200529
C	-1.245354	0.650372	-0.066128
N	-2.608849	1.226986	-0.235707
H	-2.826698	1.198486	-1.240540
H	-2.660099	2.201416	0.083538
H	1.441126	-0.334903	1.506219
H	1.444020	0.304861	-1.137017
H	-3.322683	0.689818	0.270189
H	-0.898326	0.772339	2.185614
H	0.745297	-1.290658	-0.942931

AP134a$^+$

E = –265.940469

N	-1.421265	-0.731071	-0.032392
C	-0.214702	-1.180445	-0.015312
C	0.792938	-0.094471	-0.006738
C	-0.043379	1.203197	-0.022331
C	-1.490305	0.756974	-0.039065
N	-2.523614	1.449006	-0.054953
H	-0.009590	-2.247284	-0.008559
H	1.458858	-0.213741	-0.870072
H	1.436814	-0.207008	0.874042
H	0.155756	1.818562	-0.900770
H	0.133365	1.825212	0.856219
H	-2.249162	-1.325473	-0.040613
H	-3.417442	0.959171	-0.064447

AP134b$^+$

E = –265.947628

N	-1.427099	-0.687955	-0.023798
C	-0.232332	-1.162270	-0.015111
C	0.793854	-0.088702	-0.010204
C	-0.028630	1.222363	-0.017834
C	-1.481109	0.776078	-0.026676
N	-2.612270	1.302692	-0.035065
H	-0.047030	-2.231928	-0.012051
H	1.451269	-0.209721	-0.879027
H	1.439832	-0.206742	0.867559
H	0.178023	1.833285	-0.898213
H	0.166384	1.836217	0.863162
H	-2.289111	-1.233892	-0.028547
H	-2.600742	2.322546	-0.036746

AP346$^+$

E = –265.964717

N	-1.482127	-0.703619	-0.033221
C	-0.258215	-1.136607	-0.016325
C	0.809692	-0.092705	-0.006552
C	-0.015585	1.208458	-0.021544
C	-1.417478	0.669102	-0.037218
N	-2.518505	1.359782	-0.053433
H	-0.064546	-2.206112	-0.010149
H	1.468928	-0.207893	-0.873555
H	1.446820	-0.201714	0.877613
H	0.172012	1.829507	-0.901918
H	0.149935	1.835834	0.858769
H	-3.410959	0.873434	-0.062919
H	-2.524062	2.372543	-0.057048

Table S2. DFT-calculated enthalpies (H_{298} in Hartree) and Gibbs energies (G_{298} in Hartree) including zero-point energies and thermal corrections, and entropies (S_{298} in cal mol^{-1} K^{-1}) for neutral and ionic 2-aminopyrrole.

Isomer	H_{298}	G_{298}	S_{298}
**AP16**	–265.497485	–265.533623	76.059
**AP26**	–265.469356	–265.503750	72.387
**AP36**	–265.488971	–265.523557	72.791
**AP46**	–265.481590	–265.516144	72.727
**AP56**	–265.490873	–265.525212	72.272
**IP13a**	–265.484524	–265.520227	75.142
**IP13b**	–265.486792	–265.521888	73.866
**IP15a**	–265.485424	–265.519897	72.554
**IP15b**	–265.486495	–265.521133	72.860
**IP34a**	–265.486707	–265.521655	73.553
**IP34b**	–265.481419	–265.516296	73.406
**AP1a$^-$**	–264.907099	–264.941596	72.605
**AP1b$^-$**	–264.911584	–264.946425	73.329
**AP2a$^-$**	–264.855357	–264.889913	72.730
**AP2b$^-$**	–264.854248	–264.888846	72.818
**AP3a$^-$**	–264.922108	–264.956149	71.645
**AP3b$^-$**	–264.915413	–264.949543	71.833
**AP4a$^-$**	–264.894068	–264.928555	72.584
**AP4b$^-$**	–264.892371	–264.926794	72.451
**AP5a$^-$**	–264.910649	–264.944410	71.055
**AP5b$^-$**	–264.903028	–264.936928	71.348
**AP6$^-$**	–264.929350	–264.964182	73.310
**AP116$^+$**	–265.807938	–265.842940	73.669
**AP126$^+$**	–265.826789	–265.861576	73.216
**AP136$^+$**	–265.858393	–265.893035	72.911
**AP146$^+$**	–265.831544	–265.866510	73.593
**AP156$^+$**	–265.867574	–265.902215	72.908
**AP166$^+$**	–265.824331	–265.859927	74.919
**AP266$^+$**	–265.807336	–265.842142	73.255
**AP366$^+$**	–265.804389	–265.839632	74.175
**AP466$^+$**	–265.810708	–265.846243	74.790
**AP566$^+$**	–265.813068	–265.848142	73.821
**AP134a$^+$**	–265.823288	–265.858546	74.206
**AP134b$^+$**	–265.829985	–265.864980	73.654
**AP346$^+$**	–265.846877	–265.881736	73.367

Table S3. Parameters, taken from Refs [52,53], used in Equation (2) for estimations of structural descriptors, HOMED5 for the five-membered ring and HOMED6 for the entire molecule of 2-aminopyrole isomers investigated in this work.

**Bond**	R_o	α_5	α_6
CC	1.3943	78.34	88.09
CN	1.3342	81.98	91.60

Scheme S1. Resonance structures for 2-aminopyrrole showing n-π conjugation between heavy atoms. Two labile protons at acid sites (:NH and/or $^+$NH) are indicated in bold red. During intramolecular proton-transfer equilibria (prototropy) the tautomeric protons move to base sites (:C$^-$) leading to eight tautomers given in Figure 2. The number of prototropic tautomers (eight) depend on both the number of labile protons (two) and the number of conjugated sites (six). This rule is a particular property of tautomeric systems.

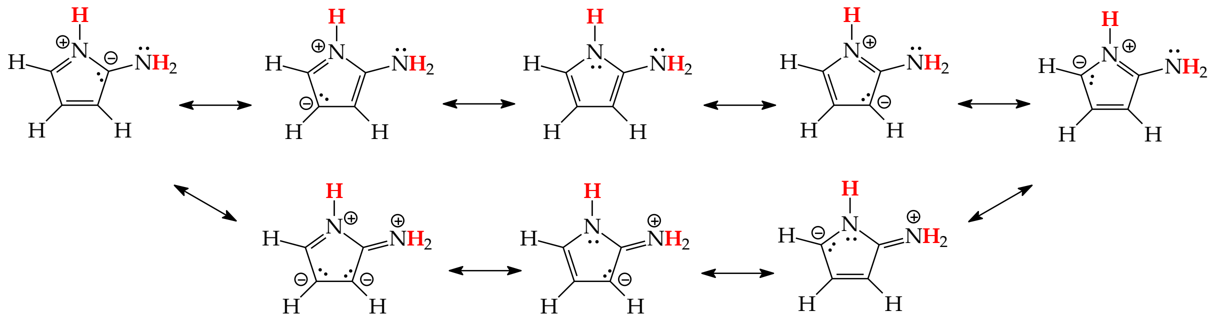

Scheme S2. Resonance structures showing n-π and/or σ-π conjugation (called hyperconjugation) for imino tautomers of 2-aminopyrrole, explaining isomerism about partially double C2=N6H, and also confirming the syn- (**a**) and anti-periplanar (**b**) positions possible for exo imino H vis-à-vis ring N1. Two labile protons are indicated in bold red.

n-π conjugation

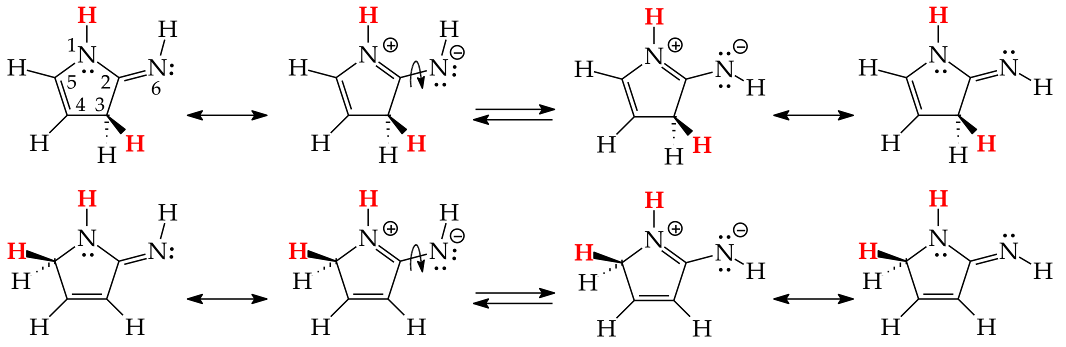

σ-π conjugation

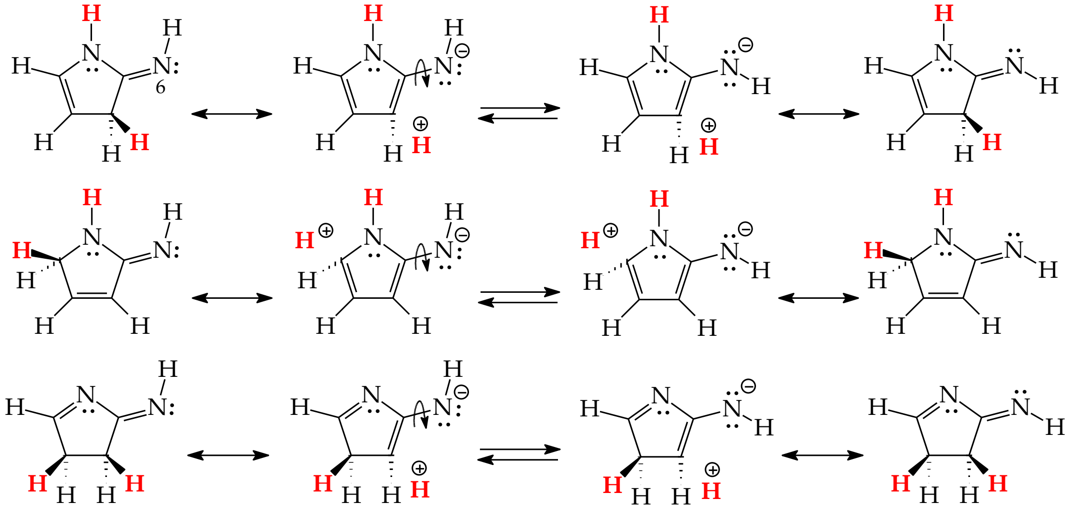

(**a**) (**b**)
